# Factors Affecting Carotid Artery Stenosis in the Elderly Living at High Altitudes

**DOI:** 10.7759/cureus.47048

**Published:** 2023-10-15

**Authors:** Abdullah Sukun, Canver Önal

**Affiliations:** 1 Radiology, Baskent University Alanya Research and Application Center, Antalya, TUR; 2 Radiology, Kars Harakani State Hospital, Kars, TUR

**Keywords:** carotid artery stenosis, eldelry, carotid artery disease, carotid doppler, carotid intima-media thickness (cimt), effect of high altitude

## Abstract

Background: Factors affecting carotid artery stenosis have been generally investigated. However, considering the protective effect of altitude, studies on elderly individuals at high altitudes are few. Our aim is to investigate the systematic adaptive changes caused by high-altitude exposure through the causes of carotid artery stenosis.

Materials and methods: Carotid Doppler ultrasound was performed on 250 patients over the age of 50 years. The patients' age, gender, height, weight, smoking history, place of residence, and presence of comorbidities were questioned. Those with diabetes, hypertension, hyperlipidemia, and coronary disease were excluded from the study. Those who did not reside at high altitudes were excluded from the study. One hundred and thirty-five patients were included in the study. Carotid artery Doppler findings and biochemical parameters were recorded. Factors affecting stenosis in the carotid arteries were compared.

Results: In our study, the factors affecting carotid stenosis were determined to be age, gender, presence of plaque, plaque type, and mean carotid intima-media thickness (CIMT). The mean CIMT of the patients was calculated as 0.71±0.14 mm. The mean CIMT measurement level was significantly higher in patients with plaque in the carotid artery (p<0.05). According to receiver operating characteristic curves, CIMT values higher than 0.72 mm increase plaque and stenosis formation in the common carotid artery (CCA) and internal carotid artery (ICA) in elderly people living at high altitudes (p<0.05, area under the curve [AUC]: 0.71-0.83).

Conclusions: The most important factors affecting carotid stenosis in individuals living at high altitudes are age, gender, CIMT, plaque, and plaque type. When soft plaques were detected, the risk of stenosis was found to be higher than in calcific plaques. Additionally, the number of factors affecting stenosis was greater on the left (ICA). This suggests that the left carotid system is more vulnerable. It can be said that in elderly individuals living at high altitudes, a CIMT higher than 0.72 mm is a cutoff value in the presence of plaque and stenosis in the carotid arteries.

## Introduction

Endothelial function at high altitudes has only been measured in populations genetically adapted to chronic hypoxia. At high altitudes, endothelial dysfunction was higher in genetically unadapted individuals and in individuals with risk factors such as diabetes, obesity, and hypertension. The vasodilatory response of arteries was found to be better in those living at high altitudes [1]. The hypoxic conditions caused by high altitude are believed to provide a protective effect against cardiovascular and metabolic diseases compared to those living at sea level. However, in the case of chronic hypoxia at high altitude, the chronic mountain disease known as hypererythrocytosis, cardiovascular risk was reported to be 3.63 times higher in individuals with cardiovascular disease than in normal individuals [2]. In the Tibetan migrant population living in high-altitude areas, women were found to have a higher prevalence of obesity and metabolic syndrome than men. There was also no association between diabetes and obesity [3]. It has been emphasized that hypoxemia at high altitudes is also evident in high body mass index (BMI), older age, male gender, and especially in the winter months [4]. The effect of altitude on hypertension was investigated in three populations living at different altitudes in Tibet. Among 1631 participants, the prevalence of hypertension decreased with increasing altitude in individuals living at low, medium, and high altitudes. It was 40.6% at low altitude, 32.5% at medium altitude, and 20.4% at high altitude. Increasing altitude decreased BMI, and thus the prevalence of hypertension decreased [5]. Those living at high altitudes had higher muscle density in the gluteus maximus and L2 trunk muscles compared to sea level, suggesting that living at a higher altitude may be beneficial for muscle quality [6]. Obesity and metabolic syndrome are risk factors for cardiovascular diseases. In a study of 10,318 participants, participants were divided into three different altitude groups: those living at altitudes lower than 500 meters, between 500 and 1500 meters, and those living at altitudes higher than 1500 meters. The prevalence of overweight was 39.5%, obesity was 22.3%, and metabolic syndrome was 31.2%. The prevalence of obesity and metabolic syndrome was higher in low-altitude areas [7]. In a study investigating the long-term effects of high altitude on choroidal thickness, it was observed that choroidal thickness was lower at high altitude. It has been emphasized that systemic adaptive changes due to chronic high-altitude exposure may cause structural changes in the choroidal vascular network [8].

The aim of our study is to investigate the factors affecting carotid artery stenosis in elderly people residing at high altitudes.

## Materials and methods

**Figure 1:**
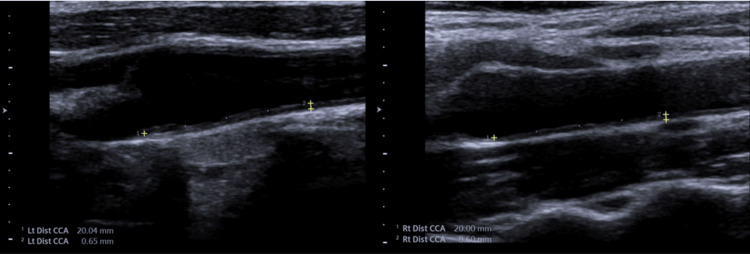
Intima-Media Thickness Measurements From Bilateral Common Carotid Artery

This study was approved by the Kafkas University Clinical Research and Ethics Committee (date: May 26, 2021, decision no. 06/15). All procedures for studies involving human participants were carried out in accordance with the 1964 Declaration of Helsinki. The authors collected data from the archives of Kars Harakani State Hospital. Research applications from this hospital are made to the Kafkas University Clinical Research and Ethics Board in the same province. Two hundred and fifty patients over the age of 50 who had carotid artery Doppler ultrasounds were performed. Patients with diabetes (23), hypertension (48), hyperlipidemia (14), and coronary artery disease (15) were excluded from the study. Individuals residing in Kars are defined as those living at high altitudes. Kars city center is located at an altitude of 1760 meters. The average altitude of the province is 1971 meters. Thirty-five percent of the province's surface area is at an altitude of 2000-2500 meters and 10% is at an altitude of 2500-3000 meters. In addition, 10 patients not residing at Kars were excluded. One hundred and thirty-five patients were included in the study. Age, gender, height, weight, BMI, smoking status, and number of pack-years in patients who smoked were recorded. Carotid Doppler ultrasonography was performed with a Toshiba Aplio 500 (Toshiba Medical Systems Corporation, Otawara, Japan). The common carotid artery (CCA) and internal carotid artery (ICA) were evaluated for stenosis and plaque using a 7.5-10 MHz probe while the patients were in supine positions, and the findings were recorded. Right and left intima-media thickness (IMT) were measured 2 cm proximal to the CCA bifurcation. Figure 1 shows the IMT measurement. The mean carotid intima-media thickness (CIMT) was also calculated from the right and left IMTs. The plaque was recorded binomially as present or absent. Patients with plaque were divided into two groups: calcific and soft plaque. Stenosis over 50% in the arteries was considered significant. The percentage of stenosis is indicated. The blood and lipid profiles of the patients were also evaluated. Red blood cell (RBC), hemoglobin (HGB), platelet (PLT), high-density lipoprotein (HDL), low-density lipoprotein (LDL), total cholesterol (TC), and triglyceride (TG) values were recorded.

Statistical analyses

Statistical analyses were performed with the Statistical Package for the Social Sciences (SPSS) version 25.0 (IBM Corp, Armonk, NY) program. The conformity of the variables to a normal distribution was examined by the Shapiro-Wilk test. Mean, standard deviation, median, minimum, and maximum values were used for descriptive analyses. The Mann-Whitney U test was used to evaluate variables that did not show a normal distribution between the two groups. Frequency and percentage values of the variables were used when presenting categorical variables. The relationships between categorical variables were analyzed by the Fisher-Freeman-Halton exact test. Differences between groups were determined by the Dunn-Bonferroni test. Receiver operating characteristic (ROC) analysis was performed to determine a threshold value for the discriminability of the mean CIMT measurement in the prediction of stenosis and plaque. When determining the threshold value, the cutoff point with the highest sensitivity and selectivity was taken. The area under the curve is interpreted as having excellent discrimination power between 1.0 and 0.90, good between 0.90 and 0.80, fair between 0.80 and 0.70, poor between 0.70 and 0.60, and almost no discriminatory power between 0.60 and 0.50. Multiple logistic regression analyses were performed to determine the independent variables affecting the stenosis-dependent variable. p-Values below 0.05 were considered statistically significant results.

## Results

A total of 135 patients were included in the study to determine the factors that most affect carotid stenosis in elderly patients at high altitudes. 60.74% of the patients were female, with a mean age of 66.78±9.65 years. 23.7% of the patients were smokers, and 21.5% of the patients had the right CCA plaque, 15.6% had right ICA plaque, 24.4% had the left CCA plaque, and 19.2% had the left ICA plaque. Right CCA stenosis was found in 5.9%, right ICA stenosis in 7.4%, left CCA stenosis in 9.6%, and left ICA stenosis in 9.6% of the patients. The mean right IMT measurement was 0.71±0.13 mm, and the mean left IMT measurement was 0.73±0.14 mm. Other clinical descriptive statistics of the patients are given in Table 1.

**Table 1:** Descriptive Statistics of the Patients CIMT: carotid intima-media thickness; BMI: body mass index; RBC: red blood cell; HGB: hemoglobin; PLT: platelet; HDL: high-density lipoprotein; LDL: low-density lipoprotein; TC: total cholesterol; TG: triglyceride; CCA: common carotid artery; ICA: internal carotid artery.

Variables		n or mean±SD	% or median (min-max)
Gender	Male	53	(39.26)
	Female	82	(60.74)
Age (years)		66.78±9.65	66 (50-91)
Weight (kg)		73.51±13.18	72 (36-117)
Height (m)		1.6±0.09	1.6 (1.42-1.85)
BMI (kg/m²)		28.29±4.76	28 (17-44)
Class	Underweight	2	(1.48)
	Normal	27	(20.00)
	Overweight	55	(40.74)
	Obese	48	(35.56)
	Morbid	3	(2.22)
Smoking	Negative	103	(76.30)
	Positive	32	(23.70)
Smoking Package Year		38.34±31.28	30 (2-120)
Residence (year)		64.97±13.31	65 (24-91)
Right Intima-Media Thickness (mm)		0.71±0.13	0.7 (0.5-1.1)
Right CCA Plaque	Negative	106	(78.52)
	Positive	29	(21.48)
Right CCA Plaque Type	Negative	106	(78.52)
	Soft	10	(7.41)
	Calcified	19	(14.07)
Right CCA Stenosis	Negative	127	(94.07)
	Positive	8	(5.93)
Right CCA Stenosis Rate		2.13±8.76	0 (0-47)
		35.88±9.28	35.5 (20-47)
Right ICA Plaque	Negative	114	(84.44)
	Positive	21	(15.56)
Right ICA Plaque Type	Negative	114	(84.44)
	Soft	7	(5.19)
	Calcified	14	(10.37)
Right ICA Stenosis	Negative	125	(92.59)
	Positive	10	(7.41)
Right CCA Stenosis Rate		3.58±13.34	0 (0-72)
		48.3±15.79	51.5 (21-72)
Left Intima-Media Thickness (mm)		0.73±0.14	0.7 (0.5-1.2)
Left CCA Plaque	Negative	102	(75.56)
	Positive	33	(24.44)
Left CCA Plaque Type	Negative	102	(75.56)
	Soft	15	(11.1)
	Calcified	18	(13.33)
Left CCA Stenosis	Negative	122	(90.37)
	Positive	13	(9.63)
Left CCA Stenosis Rate		0.35±1.16	0 (0-6)
		3.62±1.50	3 (2-6)
Left ICA Plaque	Negative	109	(80.74)
	Positive	26	(19.26)
Left CCA Plaque Type	Negative	109	(80.74)
	Soft	12	(8.89)
	Calcified	14	(10.37)
Left ICA Stenosis	Negative	122	(90.37)
	Positive	13	(9.63)
Left ICA Stenosis Rate		4.52±14.87	0 (0-79)
		46.92±17.69	50 (10-79)
Mean CIMT (mm)		0.71±0.14	0.7 (0-1.15)
RBC (10^6^/mL)		4.96±0.55	5 (2.9-6.3)
HGB (g/dL)		14.67±3.46	14.5 (10.3-49.8)
PLT (10^3^/mL)		247.25±64.51	247 (82-442)
HDL (mg/dL)		48.38±12.35	48.38 (24-85)
HDL/LDL		0.47±0.19	0.43 (0.21-1.09)
LDL (mg/dL)		112.32±34.34	112.32 (44.4-227.4)
TC (mg/dL)		190.31±41.89	190.31 (96-306)
TG (mg/dL)		149.66±77.79	140 (34-518)

There was no significant relationship between right CCA stenosis and demographic variables (p>0.05). The rates of CCA plaque, CCA plaque type, and ICA stenosis were significantly higher in patients with right CCA stenosis (p<0.05). In addition, the mean CIMT measurement level in patients with right CCA stenosis (M=0.8) was significantly higher than in patients without stenosis (M=0.7) (p=0.033) (Tables 2, 3).

**Table 2:** Variables Affecting Right CCA Stenosis Ki-Kare test. CCA: common carotid artery; ICA: internal carotid artery. *p<0.05.

Variables		Right CCA Stenosis				p-Value
		Negative		Positive		
		n	%	n	%	
Gender	Male	49	(38.58)	4	(50.00)	0.711
	Female	78	(61.42)	4	(50.00)	
Body Mass Index Class	Underweight	2	(1.57)	0	(0.00)	0.856
	Normal	26	(20.47)	1	(12.50)	
	Overweight	52	(40.94)	3	(37.50)	
	Obese	44	(34.65)	4	(50.00)	
	Morbid	3	(2.36)	0	(0.00)	
Smoking	Negative	98	(77.17)	5	(62.50)	0.394
	Positive	29	(22.83)	3	(37.50)	
Right CCA Plaque	Negative	106	(83.46)	0	(0.00)	<0.001*
	Positive	21	(16.54)	8	(100.00)	
Right CCA Plaque Type	Negative	106	(83.46)	0	(0.00)	<0.001*
	Soft	6	(4.72)	4	(50.00)	
	Calcified	15	(11.81)	4	(50.00)	
Right ICA Plaque	Negative	109	(85.83)	5	(62.50)	0.109
	Positive	18	(14.17)	3	(37.50)	
Right ICA Plaque Type	Negative	109	(85.83)	5	(62.50)	0.134
	Soft	6	(4.72)	1	(12.50)	
	Calcified	12	(9.45)	2	(25.00)	
Right ICA Stenosis	Negative	120	(94.49)	5	(62.50)	0.014*
	Positive	7	(5.51)	3	(37.50)	

**Table 3:** Variables Affecting Right CCA Stenosis Mann-Whitney U test. RBC: red blood cell; HGB: hemoglobin; PLT: platelet; HDL: high-density lipoprotein; LDL: low-density lipoprotein; TC: total cholesterol; TG: triglyceride; RIMT: right intima-media thickness; CIMT: carotid intima-media thickness; CCA: common carotid artery; ICA: internal carotid artery. *p<0.05.

Variables		Right CCA Stenosis				p-Value
		Negative		Positive		
		Mean±SD	Median (Min-Max)	Mean±SD	Median (Min-Max)	
Age (years)		66.49±9.53	66 (49-91)	71.38±11.12	73.5 (58-85)	0.198
Weight (kg)		73.47±13.14	72 (36-117)	74.13±14.60	72 (57-98)	0.985
Height (m)		1.6±0.08	1.6 (1.42-1.84)	1.58±0.12	1.55 (1.47-1.85)	0.190
Body Mass Index (kg/m²)		28.24±4.82	28 (17-44)	29.13±3.87	29.5 (24-36)	0.522
Smoking Package Year		36.79±31.47	30 (2-120)	53.33±30.55	60 (20-80)	0.317
Residence		64.57±13.37	65 (24-91)	71.38±11.12	73.5 (58-85)	0.172
RIMT		0.71±0.14	0.7 (0.5-1.1)	0.76±0.09	0.8 (0.6-0.9)	0.089*
Right ICA Stenosis Rate		46±9.24	51 (33-55)	53.67±28.36	68 (21-72)	0.425
Mean CIMT		0.71±0.14	0.7 (0-1.15)	0.79±0.08	0.8 (0.65-0.9)	0.033*
RBC (10^6^/mL)		4.98±0.52	5 (3.7-6.3)	4.64±0.82	4.75 (2.9-5.7)	0.258
HGB (g/dL)		14.72±3.54	14.6 (10.4-49.8)	14±1.74	14.35 (10.3-15.8)	0.618
PLT (10^3^/mL)		246.19±65.43	246 (82-442)	264.13±47.51	269.5 (190-340)	0.344
HDL (mg/dL)		48.56±12.54	48.38 (24-85)	45.47±8.85	48.19 (30-59)	0.560
HDL/LDL		0.48±0.20	0.43 (0.21-1.09)	0.42±0.12	0.39 (0.29-0.61)	0.631
LDL (mg/dL)		112.07±34.07	112.32 (44.4-227.4)	116.23±40.71	122.76 (55.4-171.2)	0.689
TC (mg/dL)		190.28±41.35	190.31 (100-306)	190.66±53.06	198.65 (96-253)	0.744
TG (mg/dL)		149.39±77.97	140 (34-518)	153.91±79.86	149.66 (53-297)	0.758

The age of patients with right ICA stenosis (M=74.5) was higher than that of patients without stenosis (M=65) (p=0.010). The rates of CCA plaque, ICA plaque, and ICA plaque type were significantly higher in patients with right ICA stenosis (p<0.05). In addition, right IMT and mean CIMT measurement levels were significantly higher in patients with right ICA stenosis than in patients without stenosis (p=0.042 and p=0.024, respectively). The TG measurement level was lower in patients with right ICA stenosis than in patients without stenosis (p=0.038) (Tables 4, 5).

**Table 4:** Variables Affecting Right ICA Stenosis Ki-Kare Test. CCA: common carotid artery; ICA: internal carotid artery. *p<0.05.

Variables		Right ICA Stenosis				p-Value
		Negative		Positive		
		n	%	n	%	
Gender	Male	47	(37.60)	6	(60.00)	0.190
	Female	78	(62.40)	4	(40.00)	
Body Mass Index Class	Underweight	2	(1.60)	-	-	0.797
	Normal	25	(20.00)	2	(20.00)	
	Overweight	52	(41.60)	3	(30.00)	
	Obese	43	(34.40)	5	(50.00)	
	Morbid	3	(2.40)	-	-	
Smoking	Negative	96	(76.80)	7	(70.00)	0.701
	Positive	29	(23.20)	3	(30.00)	
Right CCA Plaque	Negative	101	(80.80)	5	(50.00)	0.037*
	Positive	24	(19.20)	5	(50.00)	
Right CCA Plaque Type	Negative	101	(80.80)	5	(50.00)	0.056
	Soft	8	(6.40)	2	(20.00)	
	Calcified	16	(12.80)	3	(30.00)	
Right ICA Plaque	Negative	114	(91.20)	-	-	<0.001*
	Positive	11	(8.80)	10	(100.00)	
Right ICA Plaque Type	Negative	114	(91.20)	-	-	<0.001*
	Soft	1	(0.80)	6	(60.00)	
	Calcified	10	(8.00)	4	(40.00)	

**Table 5:** Variables Affecting Right ICA Stenosis Mann-Whitney U Test. RBC: red blood cell; HGB: hemoglobin; PLT: platelet; HDL: high-density lipoprotein; LDL: low-density lipoprotein; TC: total cholesterol; TG: triglyceride; CCA: common carotid artery; ICA: internal carotid artery; RIMT: right  intima-media thickness; CIMT: carotid intima-media thickness. *p<0.05.

Variables		Right ICA Stenosis				p-Value
		Negative		Positive		
		Mean±SD	Median (Min-Max)	Mean±SD	Median (Min-Max)	
Age (years)		66.19±9.60	65 (49-91)	74.1±7.29	74.5 (59-85)	0.010*
Weight (kg)		73.53±13.51	72 (36-117)	73.3±8.42	72 (60-92)	0.893
Height (m)		1.6±0.09	1.6 (1.42-1.85)	1.61±0.09	1.6 (1.47-1.73)	0.674
Body Mass Index (kg/m²)		28.31±4.86	28 (17-44)	28±3.40	29 (21-31)	0.909
Smoking Package Year		39.66±32.09	30 (2-120)	25.67±22.05	20 (7-50)	0.580
Residence		64.49±13.26	65 (24-91)	71±13.06	74.5 (40-85)	0.056
RIMT		0.7±0.13	0.7 (0.5-1.1)	0.82±0.19	0.8 (0.6-1.1)	0.042*
Right CCA Stenosis Rate		35.8±10.69	36 (20-47)	36±8.54	35 (28-45)	0.881
Mean CIMT		0.7±0.13	0.7 (0-1.05)	0.85±0.19	0.8 (0.65-1.15)	0.024*
RBC (10^6^/mL)		4.98±0.55	5 (2.9-6.3)	4.76±0.46	4.9 (4.1-5.4)	0.217
HGB (g/dL)		14.72±3.57	14.6 (10.3-49.8)	14.1±1.50	13.85 (12.2-16.5)	0.434
PLT (10^3^/mL)		248.59±65.11	247 (82-442)	230.5±56.78	233.5 (137-312)	0.462
HDL (mg/dL)		48.5±12.68	48.38 (24-85)	46.84±7.03	49.19 (35-59)	0.946
HDL/LDL		0.48±0.20	0.43 (0.21-1.09)	0.41±0.13	0.39 (0.22-0.61)	0.305
LDL (mg/dL)		111.17±32.98	112.32 (44.4-196.2)	126.69±48.14	121.29 (74.8-227.4)	0.403
TC (mg/dL)		190.15±41.18	190.31 (96-306)	192.23±52.42	189.15 (140-299)	0.807
TG (mg/dL)		153.29±79.24	144 (34-518)	104.23±33.14	108.5 (53-149.66)	0.038

The age of patients with left CCA stenosis (M=74) was higher than that of patients without stenosis (M=65) (p=0.048). The rates of CCA plaque, CCA plaque type, and ICA stenosis were significantly higher in patients with left CCA stenosis (p<0.05). In addition, patients with left CCA stenosis had significantly higher left IMT, left ICA stenosis (%), and mean CIMT measurements than patients without stenosis (p<0.05) (Tables 6, 7).

**Table 6:** Variables Affecting Left CCA Stenosis Ki-Kare Test. CCA: common carotid artery; ICA: internal carotid artery. *p<0.05.

Variables		Left CCA Stenosis				p-Value
		Negative		Positive		
		n	%	n	%	
Gender	Male	46	(37.70)	7	(53.85)	0.257
	Female	76	(62.30)	6	(46.15)	
Body Mass Index Class	Underweight	2	(1.64)	-	-	0.920
	Normal	24	(19.67)	3	(23.08)	
	Overweight	49	(40.16)	6	(46.15)	
	Obese	44	(36.07)	4	(30.77)	
	Morbid	3	(2.46)	-	-	
Smoking	Negative	95	(77.87)	8	(61.54)	0.188
	Positive	27	(22.13)	5	(38.46)	
Left CCA Plaque	Negative	102	(83.61)	-	-	<0.001*
	Positive	20	(16.39)	13	(100.00)	
Left CCA Plaque Type	Negative	102	(83.61)	-	-	<0.001*
	Soft	5	(4.10)	10	(76.92)	
	Calcified	15	(12.30)	3	(23.08)	
Left ICA Plaque	Negative	101	(82.79)	8	(61.54)	0.130
	Positive	21	(17.21)	5	(38.46)	
Left ICA Plaque Type	Negative	101	(82.79)	8	(61.54)	0.070
	Soft	9	(7.38)	3	(23.08)	
	Calcified	12	(9.84)	2	(15.38)	
Left ICA Stenosis	Negative	114	(93.44)	8	(61.54)	0.003*
	Positive	8	(6.56)	5	(38.46)	

**Table 7:** Variables Affecting Left CCA Stenosis Mann-Whitney U Test. RBC: red blood cell; HGB: hemoglobin; PLT: platelet; HDL: high-density lipoprotein; LDL: low-density lipoprotein; TC: total cholesterol; TG: triglyceride; CCA: common carotid artery; ICA: internal carotid artery; LIMT: left  intima-media thickness; CIMT: carotid intima-media thickness. *p<0.05.

Variables		Left CCA Stenosis				p-Value
		Negative		Positive		
		Mean±SD	Median (Min-Max)	Mean±SD	Median (Min-Max)	
Age (years)		66.24±9.54	65 (49-91)	71.85±9.62	74 (55-85)	0.048*
Weight (kg)		73.3±13.07	72 (36-117)	75.46±14.51	73 (57-102)	0.774
Height (m)		1.6±0.09	1.6 (1.42-1.84)	1.6±0.10	1.6 (1.47-1.85)	0.681
Body Mass Index (kg/m²)		28.2±4.73	28 (17-44)	29.15±5.13	28 (24-39)	0.785
Smoking Package Year		36.7±32.44	24 (2-120)	47.2±25.08	50 (16-80)	0.310
LIMT		0.71±0.13	0.7 (0.5-1.1)	0.89±0.16	0.8 (0.7-1.2)	0.000*
Left ICA Stenosis Rate		38.87±16.36	37.5 (10-62)	59.8±11.58	54 (50-79)	0.048*
Mean CIMT		0.7±0.14	0.7 (0-1.1)	0.85±0.12	0.85 (0.7-1.15)	<0.001*
RBC (10^6^/mL)		4.97±0.53	5 (3.7-6.3)	4.88±0.69	5 (2.9-5.7)	0.997
HGB (g/dL)		14.72±3.61	14.55 (10.4-49.8)	14.22±1.55	14.4 (10.3-16.2)	0.814
PLT (10^3^/mL)		248.04±64.06	251 (82-442)	239.85±70.93	220 (127-376)	0.586
HDL (mg/dL)		48.98±12.63	48.38 (24-85)	42.73±7.41	48 (30-52)	0.085
HDL/LDL		0.48±0.20	0.43 (0.21-1.09)	0.39±0.10	0.41 (0.26-0.55)	0.139
LDL (mg/dL)		111.78±34.31	112.32 (44.4-227.4)	117.38±35.58	112.32 (55.4-191.2)	0.533
TC (mg/dL)		190.07±41.70	190.31 (100-306)	192.53±45.28	190.31 (96-286)	0.849
TG (mg/dL)		147.7±77.60	135.5 (34-518)	167.97±80.32	149.66 (53-328)	0.286

The age of the patients with left ICA stenosis (M=76) was higher than that of the patients without ICA stenosis (M=65) (p=0.001). The rates of CCA plaque, CCA plaque type, ICA plaque, and ICA plaque type were significantly higher in patients with left ICA stenosis (p<0.05). In addition, left IMT and mean CIMT measurements were significantly higher in patients with left ICA stenosis than in patients without stenosis (p<0.05) (Table 8). However, HDL measurement level was lower in patients with left ICA stenosis compared to patients without stenosis (p=0.035) (Table 9).

**Table 8:** Variables Affecting Left ICA Stenosis Ki-Kare Test. ICA: internal carotid artery; LIMT: left intima-media thickness. *p<0.05.

Variables		Left ICA Stenosis				p-Value
		Negative		Positive		
		n	%	n	%	
Gender	Male	45	(36.89)	8	(61.54)	0.084
	Female	77	(63.11)	5	(38.46)	
Body Mass Index Class	Underweight	2	(1.64)	-	-	1.000
	Normal	24	(19.67)	3	(23.08)	
	Overweight	50	(40.98)	5	(38.46)	
	Obese	43	(35.25)	5	(38.46)	
	Morbid	3	(2.46)	-	-	
Smoking	Negative	93	(76.23)	10	(76.92)	1.000
	Positive	29	(23.77)	3	(23.08)	
Left CCA Plaque	Negative	97	(79.51)	5	(38.46)	0.003*
	Positive	25	(20.49)	8	(61.54)	
Left CCA Plaque Type	Negative	97	(79.51)	5	(38.46)	0.004*
	Soft	11	(9.02)	4	(30.77)	
	Calcified	14	(11.48)	4	(30.77)	
Left ICA Plaque	Negative	109	(89.34)	-	-	<0.001*
	Positive	13	(10.66)	13	(100.00)	
Left ICA Plaque Type	Negative	109	(89.34)	-	-	<0.001*
	Soft	1	(0.82)	11	(84.62)	
	Calcified	12	(9.84)	2	(15.38)	

**Table 9:** Variables Affecting Left ICA Stenosis Mann-Whitney U Test. RBC: red blood cell; HGB: hemoglobin; PLT: platelet; HDL: high-density lipoprotein; LDL: low-density lipoprotein; TC: total cholesterol; TG: triglyceride; CCA: common carotid artery; ICA: internal carotid artery; LIMT: left  intima-media thickness; CIMT: carotid intima-media thickness. *p<0.05.

Variables	Left ICA Stenosis				p-Value
	Negative		Positive		
	Mean±SD	Median (Min-Max)	Mean±SD	Median (Min-Max)	
Age	65.89±9.48	65 (49-91)	75.15±7.10	76 (60-85)	0.001*
Weight	73.05±12.79	72 (36-111)	77.85±16.35	73 (57-117)	0.449
Height	1.59±0.09	1.6 (1.42-1.85)	1.63±0.09	1.65 (1.5-1.75)	0.180
BMI	28.26±4.84	28 (17-44)	28.54±4.10	28 (24-39)	0.917
Smoking Package Year	35.41±28.79	24 (2-100)	66.67±47.26	50 (30-120)	0.144
Left Intima-Media Thickness	0.71±0.13	0.7 (0.5-1.1)	0.85±0.17	0.8 (0.7-1.2)	0.004*
Left CCA Stenosis Rate	3±1.07	3 (2-5)	4.6±1.67	5 (2-6)	0.085
Mean CIMT	0.7±0.13	0.7 (0-1.1)	0.85±0.15	0.8 (0.65-1.15)	0.001*
RBC	4.95±0.55	5 (2.9-6.3)	5.11±0.47	5.2 (4.1-5.8)	0.202
HGB	14.4±1.68	14.55 (10.3-20.4)	17.26±9.88	14.4 (12.2-49.8)	0.474
PLT	249.13±64.52	252.5 (82-442)	229.62±64.18	220 (128-376)	0.243
HDL	49.05±12.35	48.38 (24-85)	42.01±10.74	42 (26-67)	0.035*
HDL/LDL	0.48±0.19	0.43 (0.21-1.09)	0.44±0.19	0.43 (0.22-0.87)	0.458
LDL	113.14±34.77	112.32 (44.4-227.4)	104.62±30.09	111 (74-154.92)	0.393
TC	192.33±42.42	190.31 (96-306)	171.28±31.86	163 (124-228)	0.064
TG	151.52±80.03	141.5 (34-518)	132.15±51.24	120 (74-240)	0.462

The mean CIMT measurement level was significantly higher in patients with plaque (p<0.05) (Table 10). 

**Table 10:** Comparison of Mean CIMT Values According to Plaque Status Mann-Whitney U test. CCA: common carotid artery; ICA: internal carotid artery; CIMT: carotid intima-media thickness. *p<0.05.

Variables		Mean CIMT		p- Value
		Mean±SD	Median (Min-Max)	
Right CCA Plaque	Negative	0.69±0.13	0.65 (0-1.1)	<0.001*
	Positive	0.82±0.12	0.8 (0.6-1.15)	
Right ICA Plaque	Negative	0.69±0.13	0.7 (0-1)	0.002*
	Positive	0.82±0.17	0.75 (0.6-1.15)	
Left CCA Plaque	Negative	0.69±0.13	0.65 (0-1)	<0.001*
	Positive	0.8±0.14	0.8 (0.5-1.15)	
Left ICA Plaque	Negative	0.69±0.13	0.7 (0-1.1)	<0.001*
	Positive	0.83±0.14	0.8 (0.6-1.15)	

We aimed to determine a threshold value for the discriminability of the mean CIMT measurement in the prediction of CCA and ICA stenosis. Here, a high mean CIMT measurement indicates that the patient has stenosis. Accordingly, the cutoff point with the highest sensitivity and selectivity for the mean CIMT measurement in right CCA stenosis is 0.725 mm. When people with a mean CIMT value above 0.725 mm are diagnosed with right CCA stenosis, the sensitivity of the mean CIMT value is 75% and the selectivity is 61.4%. The area under the ROC curve is 72.5%. Accordingly, it can be said that the discriminative power of the mean CIMT variable is moderate. The 95% confidence interval for this area was obtained as 58%-87%. The obtained area value was found to be statistically significant (p=0.033). The mean CIMT threshold values for the other stenosis variables are also shown in Table 11.

**Table 11:** ROC Analysis of Mean CIMT Value in Predicting CCA and ICA Stenosis ROC: receiver operating characteristic; CCA: common carotid artery, ICA: internal carotid artery; CIMT: carotid intima-media thickness.

Test Result Variable(s): Mean CIMT	Area	Std. Error	Asymptotic Significance	Asymptotic 95% Confidence Interval		Cutoff (mm)	Sensitivity	Specificity
				Lower Bound	Upper Bound			
Right CCA Stenosis	0.725	0.074	0.033	0.580	0.870	>0.725	75.00%	61.4%
Right ICA Stenosis	0.714	0.090	0.025	0.537	0.891	>0.725	70.00%	61.6%
Right CCA Plaque	0.796	0.044	<0.001	0.709	0.882	>0.725	82.76%	70.8%
Right ICA Plaque	0.715	0.063	0.002	0.591	0.838	>0.725	66.67%	64.0%
Left CCA Stenosis	0.838	0.044	0.000	0.751	0.925	>0.725	84.62%	63.9%
Left ICA Stenosis	0.785	0.057	0.001	0.673	0.898	>0.725	84.62%	63.9%
Left CCA Plaque	0.728	0.052	0.000	0.627	0.829	>0.725	69.70%	68.6%
Left ICA Plaque	0.769	0.051	0.000	0.670	0.868	>0.725	76.92%	67.9%

In patients with right-left CCA and ICA stenosis, the proportion of males (57.69%) was higher and the proportion of females (42.31%) was lower (p=0.032). The rates of CCA plaque, CCA plaque type, ICA plaque, and ICA plaque type were also significantly higher in patients with stenosis (p<0.001) (Table 12). Age measurement was higher in patients with stenosis (p=0.001). In addition, the mean CIMT measurement level (M=0.8) was higher in patients with stenosis than in patients without stenosis (p<0.001) (Table 13).

**Table 12:** Factors Causing Significant Stenosis in All Patients Ki-Kare Test. CCA: common carotid artery; ICA: internal carotid artery. *p<0.05.

Variables		Significant Stenosis				p-Value
		Negative		Positive		
		n	%	n	%	
Gender	Male	38	(34.86)	15	(57.69)	0.032*
	Female	71	(65.14)	11	(42.31)	
Body Mass Index Class	Underweight	2	(1.83)	-	-	0.905
	Normal	23	(21.10)	4	(15.38)	
	Overweight	44	(40.37)	11	(42.31)	
	Obese	37	(33.94)	11	(42.31)	
	Morbid	3	(2.75)	-	-	
Smoking	Negative	86	(78.90)	17	(65.38)	0.145
	Positive	23	(21.10)	9	(34.62)	
CCA Plaque	Negative	86	(78.90)	5	(19.23)	<0.001*
	Positive	23	(21.10)	21	(80.77)	
CCA Plaque Type	Negative	86	(78.90)	5	(19.23)	<0.001*
	Soft	6	(5.50)	11	(42.31)	
	Calcified	17	(15.60)	10	(38.46)	
CCA Plaque	Negative	93	(85.32)	8	(30.77)	<0.001*
	Positive	16	(14.68)	18	(69.23)	
ICA Plaque Type	Negative	93	(85.32)	8	(30.77)	<0.001*
	Soft	2	(1.83)	11	(42.31)	
	Calcified	14	(12.84)	7	(26.92)	

**Table 13:** Factors Causing Significant Stenosis in All Patients Mann-Whitney U test. RBC: red blood cell; HGB: hemoglobin; PLT: platelet; HDL: high-density lipoprotein; LDL: low-density lipoprotein; TC: total cholesterol; TG: triglyceride; CIMT: carotid intima-media thickness. *p<0.05.

Variables	Significant Stenosis				p-Value
	Negative (n=109)		Positive (n=26)		
	Mean±SD	Median (Min-Max)	Mean±SD	Median (Min-Max)	
Age (years)	65.46±9.44	65 (50-91)	72.31±8.68	74.5 (55-85)	0.001*
Weight (kg)	72.57±12.67	72 (36-111)	77.46±14.72	74 (57-117)	0.223
Height (m)	1.59±0.08	1.6 (1.42-1.84)	1.61±0.10	1.6 (1.47-1.85)	0.508
Body Mass Index (kg/m²)	28.06±4.80	28 (17-44)	29.23±4.53	28 (21-39)	0.326
Smoking Package Year	35.39±29.52	24 (2-100)	45.89±36.15	30 (7-120)	0.409
Mean CIMT	0.69±0.13	0.65 (0-1)	0.82±0.13	0.8 (0.65-1.15)	<0.001*
RBC (10^6^/mL)	4.97±0.53	5 (3.7-6.3)	4.93±0.62	5 (2.9-5.8)	0.808
HGB (g/dL)	14.45±1.69	14.6 (10.4-20.4)	15.63±7.12	14.35 (10.3-49.8)	0.986
PLT (10^3^/mL)	250.84±64.52	253 (82-442)	232.19±63.48	229.5 (127-376)	0.197
HDL (mg/dL)	49.4±12.75	48.38 (24-85)	44.07±9.52	48.19 (26-67)	0.077
HDL/LDL	0.48±0.20	0.43 (0.21-1.09)	0.42±0.15	0.4 (0.22-0.87)	0.151
LDL (mg/dL)	111.69±32.86	112.32 (44.4-196.2)	114.98±40.57	112.32 (55.4-227.4)	0.978
TC (mg/dL)	191.44±40.37	190.31 (100-306)	185.55±48.32	187.5 (96-299)	0.286
TG (mg/dL)	152.65±79.06	143 (34-518)	137.09±72.31	117 (53-328)	0.262

A regression model was created to estimate the significant effects of independent variables on the dependent variable of stenosis. Independent variables with p-value <0.20 in the single logistic regression model were included in the multiple logistic regression model. In the multiple logistic regression model, the best model was obtained by using the forward (LR) selection method (-2 Log likelihood = 64.043; Nagelkerke R square = 0.635; p<0.001). The model fit was adequate (Hosmer and Lemeshow test, p=0.753). In the estimated model, CCA plaque type and ICA plaque type independent variables contributed significantly to the model. The risk of stenosis in patients with plaque type soft was 47.7 times higher than in patients without plaque (p<0.001; 95% CI (7.617-298.998)). The risk of stenosis in patients with plaque type Ca is 14.5 times higher than in patients without plaque (p=0.005; 95% CI: 7.232-94.421). The risk of stenosis in patients with ICA plaque type soft was 175.86 times higher than in patients without plaque (p<0.001; 95% CI (15.27-2025.3)). The overall classification percentage of the estimated model was 88.9% (Table 14).

**Table 14:** Multiple Logistic Regression Analysis HDL: high-density lipoprotein; LDL: low-density lipoprotein; CCA: common carotid artery; ICA: internal carotid artery.

Variables	B	SE	Wald	df	Sig	Exp (B)	%95 CI for Exp (B)	
							Lower	Upper
CCA Plaque Type			17.389	2	0.000			
Soft	3.865	0.936	17.046	1	<0.001	47.723	7.617	298.998
Calcified	2.675	0.955	7.841	1	0.005	14.516	2.232	94.421
ICA Plaque Type			17.777	2	<0.001			
Soft	5.170	1.247	17.191	1	<0.001	175.861	15.270	2025.313
Calcified	1.024	0.814	1.584	1	0.208	2.785	0.565	13.729
HDL/LDL	-4.589	2.357	3.789	1	0.052	0.010	0.000	1.032
Constant	-2.046	1.144	3.198	1	0.074	0.129		

## Discussion

In our study investigating the factors affecting carotid stenosis in elderly patients at high altitudes, factors were evaluated bilaterally for CCA and ICA. We found that the factors affecting stenosis were associated with gender, age, plaque, plaque type, duration of high altitude, and mean IMT thickness. We also determined the mean CIMT cutoff value affecting plaque formation for bilateral CCA and ICA in individuals living at high altitudes. The risk of significant stenosis in CCA and ICA differed significantly according to plaque type.

A study in a Peruvian population living at high altitudes found a significant and negative association with the Framingham 10-year risk score in individuals living above 2500 meters, with lower mean systolic blood pressure and lower diagnoses of diabetes compared to those residing at lower altitudes [9]. In a study investigating the effects of smoking and altitude on blood 25-hydroxy vitamin D, testosterone, and carotid artery thickness, smoking significantly increased the intima-media thickness of the right and left carotid arteries at both high and low altitudes (p≤0.001). Smoking at high altitudes was reported to be associated with a significant increase in 25-hydroxy vitamin D and testosterone concentrations, whereas at low altitudes it was associated with a significant decrease in both parameters. It was emphasized that changes in these biochemical parameters should be kept in mind in the dose modification of drugs when treating patients [10]. In our study, since the altitude was kept constant, the effect of biochemical parameters on carotid stenosis was not found in individuals living at high altitudes. Although the mean number of cigarette pack-years smoked was higher in individuals with significant carotid stenosis in our study, no statistical difference was found. However, mean IMT values were significantly higher, indicating that cigarette smoking affects IMT.

Carotid atherosclerosis is a risk factor for ischemic stroke. Detection of plaques, stenosis, and IMT on carotid Doppler ultrasound can prevent further strokes. In ischemic stroke cases, IMT was found to be 0.79±0.10 mm on the right side and 0.90±0.17 mm on the left, and CIMT was found to be significantly higher in the case group than in the control group [11]. In our study, the mean CIMT thickness was found to be significantly higher in individuals with significant stenosis in the CCA and ICA (0.82±0.13). In individuals living at high altitudes, the average IMT thickness being higher than 0.725 mm was found to be a significant cutoff value for the development of plaque and stenosis in ICA and CCA.

CIMT values have been researched for many diseases in the literature. The average was found to be 0.91 mm in lacunar infarcts and 1.04 mm in non-lacunar infarcts [12]. In ischemic strokes, large vessel and small vessel disease differ in terms of CIMT. CIMT values are significantly higher in large vessel involvement (1.08 mm) than in small vessel involvement (0.92 mm) [13]. Diabetic patients were found to have increased CIMT values in parallel with more atherosclerotic changes compared to non-diabetic patients [14]. In a cohort of middle-aged women, metabolic syndrome was found to be more strongly associated with atherosclerosis as determined by CIMT than by the International Diabetes Federation definition or other definitions of metabolic health, and more strongly than BMI or waist circumference [15]. In our study, significant stenosis was compared with gender, age, and BMI. Age and gender were found to be important variables. In a systematic review and meta-analysis study including 16,179 cases and 26,120 control individuals, it was reported that a 0.12 mm increase in CIMT was associated with non-alcoholic liver disease [16]. A systematic review investigating the global variation and demographic relationship of CIMT included 49,381 individuals with a mean age of 55.6 years. The average CIMT value for the group without coronary heart disease was found to be 0.65 and showed regional differences. CIMT in Europe was detected at 0.71 mm. CIMT was found to be 0.23 mm higher in the group with coronary heart disease. In this review, it was reported that CIMT increases with age, and the average is 0.76 in the 60-69 years age range. In our study, the median and mean age were 66, and the mean IMT was found to be 0.71±0.14 and 0.69±0.13 in individuals without stenosis, which is correlated with the study results [17]. In a study investigating the characteristics of carotid plaques, the plaques, which were described as predominantly hypoechoic and soft plaques in our study, were found to be associated with cerebrovascular symptomatic individuals. Surface irregularity, plaque length, and area have been reported as other important variables of plaque morphology. However, no difference was detected between cerebrovascular symptomatic and non-symptomatic individuals in terms of plaque thickness and CIMT [18]. Carotid artery atherosclerosis is distributed asymmetrically in the carotid vessels. Plaques were more common on the left. Additionally, intraplaque bleeding and fibrous tissue were more commonly detected in the carotid plaques on the left side. It is emphasized that the plaques on the left are more vulnerable and the plaques on the right are more stable [19]. In our study, it was determined that the area under the curve of the average CIMT value in the ROC curves used to detect plaque and stenosis was greater on the left.

The limitations of our study are that it was a retrospective, single-center study, and was evaluated in a limited number of patients. Furthermore, although diseases affecting carotid stenosis were excluded, there are no data on ethnicity, lifestyle, dietary habits, or exercise that may affect stenosis. 

## Conclusions

Consequently, all variables were analyzed separately for bilateral CCA and ICA. The most important variables in all patients were age, gender, CIMT, plaque in CCA and ICA, and plaque type. When soft plaques were detected, the risk of stenosis was found to be higher than in calcific plaques. Additionally, the number of factors affecting stenosis was greater in the left ICA. This suggests that the left carotid system is more vulnerable. The CIMT value was significantly higher in patients with plaque in the carotid artery. Our results show that CIMT values higher than 0.72 mm are associated with plaque and stenosis in the carotid arteries in elderly individuals living at high altitudes.

## References

[1] Calderón-Gerstein WS, López-Peña A, Macha-Ramírez R (2017). Endothelial dysfunction assessment by flow-mediated dilation in a high-altitude population. Vasc Health Risk Manag.

[2] Corante N, Anza-Ramírez C, Figueroa-Mujíca R (2018). Excessive erythrocytosis and cardiovascular risk in Andean Highlanders. High Alt Med Biol.

[3] Lin BY, Genden K, Shen W (2018). The prevalence of obesity and metabolic syndrome in Tibetan immigrants living in high altitude areas in Ladakh, India. Obes Res Clin Pract.

[4] Vignati C, Mapelli M, Nusca B (2021). A breathtaking lift: sex and body mass index differences in cardiopulmonary response in a large cohort of unselected subjects with acute exposure to high altitude. High Alt Med Biol.

[5] Song C, Chongsuvivatwong V, Zhu Luo Bu O, Ji D, Sang Zhuo Ma B, Sriplung H (2020). Relationship between hypertension and geographic altitude: a cross-sectional survey among residents in Tibet. J Int Med Res.

[6] Liu X, Wang L, Gao M (2021). Comparison of muscle density in middle-aged and older Chinese adults between a high-altitude area (Kunming) and a low-altitude area (Beijing). Front Endocrinol (Lausanne).

[7] Pérez-Galarza J, Baldeón L, Franco OH, Muka T, Drexhage HA, Voortman T, Freire WB (2021). Prevalence of overweight and metabolic syndrome, and associated sociodemographic factors among adult Ecuadorian populations: the ENSANUT-ECU study. J Endocrinol Invest.

[8] Gok M, Karaman S, Erdem B (2022). Evaluation of macular and choroidal thickness in healthy residents living at high altitude. Indian J Ophthalmol.

[9] Hernández-Vásquez A, Vargas-Fernández R, Chacón-Diaz M (2022). Association between altitude and the Framingham risk score: a cross-sectional study in the Peruvian adult population. Int J Environ Res Public Health.

[10] Zaman GS, Alshahrani SA, Laskar NB (2022). Association of smoking with the blood concentration of 25-hydroxy vitamin D and testosterone at high and low altitudes. Int J Gen Med.

[11] Kawnayn G, Kabir H, Huq MR, Chowdhury MI, Shahidullah M, Hoque BS, Anwar MB (2023). The association of carotid plaque size, carotid intima-media thickness, resistive index, and pulsatility index with acute ischemic stroke. Cureus.

[12] Cupini LM, Pasqualetti P, Diomedi M (2002). Carotid artery intima-media thickness and lacunar versus nonlacunar infarcts. Stroke.

[13] Pruissen DM, Gerritsen SA, Prinsen TJ, Dijk JM, Kappelle LJ, Algra A (2007). Carotid intima-media thickness is different in large- and small-vessel ischemic stroke: the SMART study. Stroke.

[14] Bill O, Mazya MV, Michel P, Prazeres Moreira T, Lambrou D, Meyer IA, Hirt L (2022). Intima-media thickness and pulsatility index of common carotid arteries in acute ischaemic stroke patients with diabetes mellitus. J Clin Med.

[15] Magri CJ, Xuereb S, Xuereb RA, Fava S (2023). Metabolic health and carotid intima-media thickness: association of different definitions in women. Am J Cardiol.

[16] Khoshbaten M, Maleki SH, Hadad S, Baral A, Rocha AV, Poudel L, Abdshah A (2023). Association of nonalcoholic fatty liver disease and carotid media-intima thickness: a systematic review and a meta-analysis. Health Sci Rep.

[17] Abeysuriya V, Perera BP, Wickremasinghe AR (2022). Regional and demographic variations of carotid artery intima and media thickness (CIMT): a systematic review and meta-analysis. PLoS One.

[18] Sultan SR, Khayat M, Almutairi B (2023). B-mode ultrasound characteristics of carotid plaques in symptomatic and asymptomatic patients with low-grade stenosis. PLoS One.

[19] Selwaness M, van den Bouwhuijsen Q, van Onkelen RS (2014). Atherosclerotic plaque in the left carotid artery is more vulnerable than in the right. Stroke.

